# Detection of ovulation, a review of currently available methods

**DOI:** 10.1002/btm2.10058

**Published:** 2017-05-16

**Authors:** Hsiu‐Wei Su, Yu‐Chiao Yi, Ting‐Yen Wei, Ting‐Chang Chang, Chao‐Min Cheng

**Affiliations:** ^1^ Dept. of Obstetrics, Gynecology & Women's Health Taichung Veterans General Hospital Taichung Taiwan; ^2^ Interdisciplinary Program of Life Science National Tsing Hua University Hsinchu Taiwan; ^3^ Div. of Gynecologic Oncology, Dept. of Obstetrics and Gynecology Chang Gung Memorial Hospital and Chang Gung University and Gynecologic Cancer Research Center, Chang Gung Memorial Hospital Taoyuan Taiwan; ^4^ Institute of Biomedical Engineering, National Tsing Hua University Hsinchu Taiwan

**Keywords:** family planning, fertility window, ovulation detection

## Abstract

The ability to identify the precise time of ovulation is important for women who want to plan conception or practice contraception. Here, we review the current literature on various methods for detecting ovulation including a review of point‐of‐care device technology. We incorporate an examination of methods to detect ovulation that have been developed and practiced for decades and analyze the indications and limitations of each—transvaginal ultrasonography, urinary luteinizing hormone detection, serum progesterone and urinary pregnanediol 3‐glucuronide detection, urinary follicular stimulating hormone detection, basal body temperature monitoring, and cervical mucus and salivary ferning analysis. Some point‐of‐care ovulation detection devices have been developed and commercialized based on these methods, however previous research was limited by small sample size and an inconsistent standard reference to true ovulation.

## 1 INTRODUCTION

From the beginning of adolescence to menopause, menstruation cycles affect female health, mood, and daily living quality for a span of approximately 35–40 years in the normal population. The main purpose underlying this sophisticated, naturally designed mechanism is to provide gametes and a well‐prepared environment for embryo implantation.

Inside the ovary, multiple follicles grow under the influence of follicular stimulating hormone (FSH), which is secreted by the pituitary gland. These growing follicles secrete estrogen, which subsequently inhibits FSH secretion in a negative feedback loop involving the pituitary gland, the hypothalamus, and inhibin B.^1^, ^2^, ^3^, ^4^ By making itself more sensitive to lowered FSH level while adjacent follicles experience atresia as a result of decreasing FSH, a dominant follicle is naturally selected and allowed to grow.^5^ This dominant follicle continues to secrete estrogen. The persistent high level of estrogen induces an abrupt release of luteinizing hormone from the pituitary gland,^6^ and this hormonal surge then triggers ovulation.^7^ After ovulation, the dominant follicle transforms into a corpus luteum, which secrets estrogen and progesterone and collapses, initiating menstruation.

Detection and monitoring of ovulation has long been practiced by women pursuing or avoiding pregnancy. The fertility window begins approximately 3–5 days (sperm lifespan) before ovulation and continues to a point approximately 1–2 days (oocyte lifespan) after ovulation.^8^ Identifying this window, rather than simply identifying or detecting ovulation, is vital for encouraging or discouraging contraception. For physicians or women who wish to know if a menstrual cycle is normal or to evaluate ovarian function, a test that retrospectively confirms ovulation should suffice, but for artificial reproductive techniques, the time of ovulation and the fertility window must be defined clearly.

By performing ultrasonography, the maximum growth of the dominant follicle and the subsequent decrease in size can be observed, so the time of ovulation, which lies in between, can be determined. Because this time can be clearly defined in this manner, it is recognized as the standard reference examination for ovulation detection and is used mainly in artificial reproductive techniques. Detection of the luteinizing hormone (LH) surge, whether in serum or in urine, is very sensitive and specific for ovulation and provides great accuracy for determining conception capacity. However, because sperm ejaculated before a woman's LH surge may survive long enough to fertilize the ovum, methods that simply determine this surge are not ideal for contraception. Before the LH surge, serum estrogen level rises and several changes occur in body fluid components, including cervical mucus and saliva. Observation of these differences may provide a better view of the fertility window. Progesterone is secreted by the corpus luteum only after ovulation. Detection of progesterone or its metabolites can retrospectively confirm the occurrence of ovulation. Because progesterone causes a rise in basal body temperature (BBT), a measure of this temperature may also be useful for determining ovulation. Because the oocyte dies shortly after ovulation, methods that correlate to progesterone and its effect identify fertility window closure.

Current methods to detect ovulation and their corresponding point‐of‐care (POC) devices are reviewed in this article. Physicians and those who are pursuing or avoiding pregnancy should know the indications and limitations of each method before employing them.

## 2 CONVENTIONAL METHODS

### 2.1 Ultrasonography

**Figure 1:**
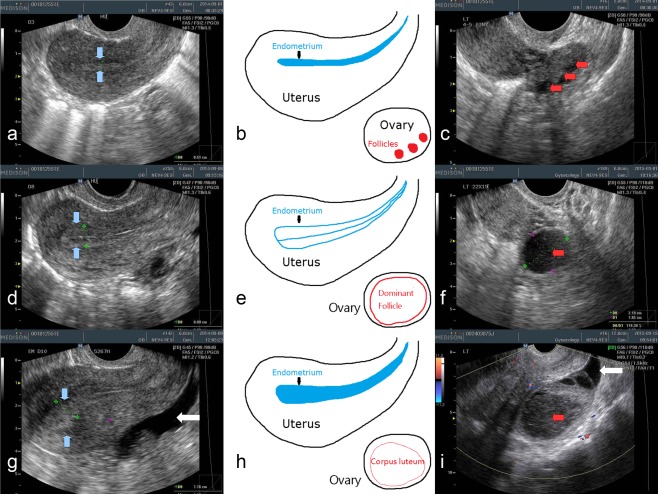
Transvaginal ultrasonography of ovary and endometrium in early follicular phase (a–c), late follicular (shortly before ovulation) phase (d–f), and post‐ovulatory luteal phase (g–i). In early follicular phase, the endometrium (between the blue arrows) just after menstruation appeared thin and homogenous (a). Multiple small follicles (red arrows) could be seen inside the ovary (c). In late follicular phase, the endometrium was thickened, and a typical “triple line” appearance could be seen (d). A dominant follicle, approximately 2 cm in diameter, is about to ovulate in late follicular phase (f). After ovulation, the endometrium becomes “luteinized,” with increased echogenicity (whitening) (g). The dominant follicle transforms into a corpus luteum (i). Note the increased echogenicity and inhomogenous content (with filament‐like structure) inside the corpus luteum compared to the dominant follicle in picture f. Free fluid can be seen in the pelvis (white arrows in pictures g and f)

Transvaginal ultrasonography can clearly define ovulation time and is recognized as the standard reference examination for detecting ovulation. It is performed by experienced technicians, radiologists, or gynecologists. Being invasive, expensive, and inconvenient, this technique is not broadly used; it is used mainly in gynecological clinics and is often performed as a step in artificial reproductive techniques. Using serial ultrasonography examinations, the time of ovulation can be determined as the point between maximum follicular diameter and follicular collapse (Figure 1). Indications of ovulation include the following:

1. Disappearance or sudden decrease in follicle size.
2. Increased echogenicity inside the follicle, indicating corpus luteum formation.
3. Free fluid in pelvis (or pouch of Douglas).
4. Replacement of “triple‐line appearance” of endometrium by homogenous, hyperechoic “luteinized” endometrium.^9^, ^10^

### 2.2 Urinary luteinizing hormone

LH belongs to a group of glycoprotein hormones that share a common α subunit, and biological specificity for each hormone is conferred by their divergent β subunit. While serum estradiol (the most potent estrogen secreted by the dominant follicle) concentration reaches a threshold level (greater than 200 pg/ml for approximately 50 hr), a positive feedback mechanism works on the hypothalamus and anterior pituitary gland, which results in an abrupt secretion of LH into bloodstream. The onset of the LH surge precedes ovulation by 35–44 hr, and the peak serum level of LH precedes ovulation by 10–12 hr.^11^, ^12^ A study of 155 cycles from 35 women demonstrated that the onset of the LH surge primarily occurs between midnight and early morning (37% between 00:00 and 04:00, 48% between 04:00 and 08:00).^13^

Detection of LH in urine using an over‐the‐counter device is much more convenient and less invasive than measuring serum LH level by multiple venipuncture. Women wishing to accurately determine their fertility window are instructed to record several menstrual cycles to estimate the possible time of ovulation. Starting from the 10th to 11th day of a new cycle (day 1 is defined as the first day of menstrual bleeding), or 4 days before the estimated ovulation day, women can test their urinary LH once or twice daily. Highly sensitive urinary LH kits detect concentrations as low as 22 mIU/ml, while natural LH surge concentration in urine ranges from 20 to 100 mIU/ml.^14^ The mean time interval after a positive urinary LH test to follicular rupture detected by sonography was reported to be 20 ± 3 hr (95% CI 14–26),^15^ and in a study focused on infertile women, sensitivity, specificity, and accuracy of the urinary LH test to detect ovulation reached 1.00, 0.25, and 0.97, respectively.^16^ Its accuracy proven, the U.S. National Academy of Clinical Biochemistry Laboratory Medicine practical guidelines recommend using the urinary LH test, because a positive result predicts ovulation within 48 hr (Strength B, level II).^17^ Several studies^18^, ^19^, ^20^, ^21^, ^22^ describe methods to detect ovulation use urinary LH surge instead of ultrasonography because it is highly accurate, inexpensive, and less invasive.

Despite positive correlations used and mentioned in the literature, there are still some indications that LH surge may not signify true ovulation. In an observational study of 43 women in which urinary LH was recorded and analyzed daily, it was found that LH surges are not of one type, and they are extremely variable. The onset of urinary LH surge was categorized into rapid‐onset type (within one day, 42.9%) and gradual‐onset type (over 2–6 days, 57.1%). Configurations of LH surge can be categorized into three types: (a) spiking (41.9%); (b) biphasic (44.2%); and, (c) plateau (13.9%).^23^ Furthermore, two (4.3%) women demonstrated LH surge without ovulation. In infertile women, premature LH surge that did not trigger ovulation was detected in 46.8% of cycles.^24^ Also, a situation reported as “luteinized unruptured follicle syndrome” was reported to occur in 10.7% of menstrual cycles in normally fertile women. Women with this syndrome have a normal LH surge, functioning corpus luteum, and menstruation, but no oocyte is released.^25^, ^26^

Because a positive urinary LH test precedes ovulation, it is theoretically helpful for timed intercourse or intrauterine insemination because the clinical pregnancy rate after a single incident of intercourse is highest from a point 2 days before ovulation to the day of ovulation.^27^ In a study mainly focused on the psychological stress in women using a urinary LH kit, a trend toward increased self‐reported pregnancy rate can be seen, but this result did not reach statistical significance (the odds of pregnancy rate in the study group were 1.77 (95% CI: 0.9992, 3.1585) compared with the control group).^28^ A 2015 Cochrane review concluded that timed intercourse using urinary hormone monitoring was associated with an increased pregnancy rate (RR 1.36, 95% CI 1.06–1.73, 3 RCTs, *n* = 1,370).^29^ A promising unpublished study that may provide a better answer to this question is the Oxford Conception Study, which targets conception rate as its primary outcome.^30^

### 2.3 Serum progesterone and urinary pregnanediol 3‐glucuronide

After ovulation, the dominant follicle turns into a corpus luteum and begins to secrete progesterone. To confirm ovulation, serum progesterone or its metabolite in urine, can be measured. A single serum progesterone level >3 ng/ml in mid‐luteal phase has been used to retrospectively detect ovulation. A recent European study proposed a random serum progesterone ≥5 ng/ml for confirming ovulation with sensitivity and specificity at 89.6% and 98.4%, respectively.^31^ The same study group also demonstrated that levels of urinary pregnanediol 3‐glucuronide (PDG), a metabolite of progesterone, measured at levels over 5 μg/ml for three consecutive days could be used as positive confirmation of ovulation with a sensitivity of 92.2% and a specificity of 100%.^32^ Nevertheless, a convenient POC device to detect urinary PDG has not yet been developed.

### 2.4 Urinary follicular stimulating hormone

Li et al. reported that peak FSH level occurred within 1 day of ultrasonography‐detected follicular collapse in 97% of menstrual cycles. The threshold value of urinary FSH was not mentioned.^33^ No further study has been published regarding this method to detect ovulation.

### 2.5 Basal body temperature

**Figure 2:**
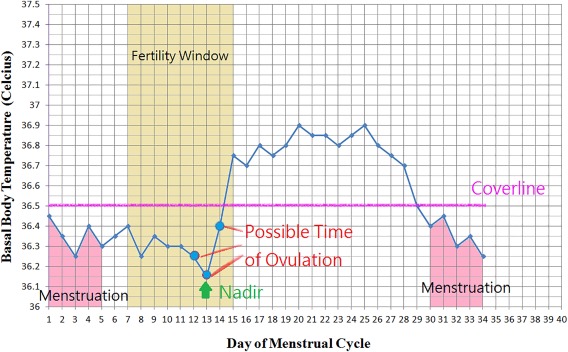
An illustration of BBT chart in degrees Celsius. This is a biphasic pattern in a normal ovulatory cycle. Ovulation can only be suggested after observing the rise and plateau of temperature above the purple “Coverline.” A drop in temperature occurs at the end of the luteal phase when progesterone decreases, which is followed by menstruation

In 1906, Theodoor Hendrik van de Velde noticed a biphasic change of BBT in women during menstruation. Monitoring of BBT has become one of the simplest and least invasive methods to detect ovulation. The rise of BBT results from the thermogenic effect of progesterone. During the follicular phase of the menstrual cycle, BBT keeps in the lower range, generally between 97.0 and 98.0°F, until approximately 1 day before ovulation, when BBT reaches its lowest point (nadir, or dip). After ovulation, the corpus luteum begins to secret progesterone. The BBT rises 0.5–1.0°F and plateaus throughout the luteal phase. In late luteal phase, when the corpus luteum regresses and serum progesterone level decreases, the BBT returns to the lower range within 1–2 days before, or just at, the onset of menstrual bleeding. This biphasic pattern of BBT retrospectively suggests ovulation (Figure 2).

Women interested in determining their fertility window are instructed to measure their oral, vaginal, or rectal temperature every day when they wake up and before any activity is initiated. Modern digital thermometers have recording abilities that make monitoring BBT more convenient. However, a traditional glass thermometer that is accurate to 1/10th of a degree is good enough for determining BBT. Women can record their BBT on specialized charts, online services, or applications on smart‐phone devices.

There are many ways to interpret BBT. In one approach, the “coverline” method, a horizontal coverline (which means a threshold temperature) is drawn on a BBT chart. When temperature above this coverline is recorded, ovulation is suggested. The coverline can be determined by adding 0.15°F to the highest temperature recorded during the first 10 days of a cycle, or by using previously recorded temperatures. Using the “three over six” rule,^34^ three temperatures are required to be 0.2°F above the highest point of the previous six temperatures with at least one of the higher temperatures being 0.4°F above the lower ones.

Factors influencing BBT include fever, alcohol, emotional or physical stress, sleep disturbance, change of room temperature, change of waking time, change of climate, and recent start or discontinuation of birth control pills or anti‐pyretics. A “GAP” technique was described in 2005 as a superior approach to the coverline method for women seeking contraception. By subtracting the BBT of a cohabiting male partner from the female partner's BBT, the temperature “gap” may be recorded. This gap is theoretically not influenced by environmental factors other than hormonal influences of the female. However, the study group was small (33 cycles), and the approach is not suitable for women who have not, or do not, live with their male partners.^35^

Interpretation of BBT is not always easy. Even for natural family planning experts, there has been poor agreement on the first day of BBT rise between different observers.^36^ It was reported that the time of ovulation determined by BBT coincided with the LH surge ± 1 day in only 17 of 77 cycles (22.1%).^37^ A review article published in 2005 concluded that monitoring BBT was no longer a good predictor of ovulation and, therefore, should not be recommended for couples seeking pregnancy.^38^ Nevertheless, BBT is still broadly used for contraception and for evaluation of ovulatory function, especially for couples who are reluctant or unable to pursue more formal and costly evaluation.

### 2.6 Cervical mucus

Cervical mucus is secreted by cervical and endocervical glands. The appearance of cervical mucus varies during different stages of the menstrual cycle. Outside of the periovulatory period, this mucus is mainly composed of high‐molecular‐weight glycoprotein (mucin), which forms a mesh‐like structure that provides a barrier to sperm and micro‐organisms. The mucus appears thick, scant, and viscous by inspection. During the periovulatory period, under the effect of estrogen, the production of acellular water increases and the production of mucin decreases. The mesh‐like structure consequently loosens and becomes highly receptive for sperm penetration.^18^, ^39^, ^40^ Women experience an increased amount of watery discharge resembling raw egg white during this period.

Observing cervical mucus is the least expensive method to detect ovulation. Women may simply observe mucus found externally at the vulva or collect vaginal mucus using their fingers. While uncomplicated, this approach has still been shown to be effective. In a small study involving 12 cycles recorded by 6 women, finding the most abundant fertile type of mucus correlated to ± 1 day of ovulation detected by ultrasonography.^41^ In a larger study involving 148 cycles recorded by 40 women, mucus sensation and characteristics yielded a 48.3% correlation to ultrasonography‐detected ovulation.^42^ In another large study, cervical mucus‐detected methodology for determining expected date of ovulation correlated to ± 1 day of ultrasonography‐detected ovulation in 160/215 cycles (74.4%).^43^ In a study involving 29 cycles recorded by 15 women, the sensitivity of detecting fertile mucus from the vulva and vagina compared to ± 1 day of ultrasonography‐detected ovulation were 75.9% and 75.9%, respectively. When the period was extended to −1 to +2 of ovulation, the sensitivities were 96.9% (vulva) and 89.6% (vagina), respectively.^44^

The combination of measuring BBT and observing cervical mucus for contraception is called the symptothermal method. Among women using this method for contraception in one study, the unintended pregnancy rate was 1.8%.^45^

### 2.7 Salivary ferning and analysis

The history of salivary ferning test can be traced back to 1969. Dr. Biel Cassal found that the arborization (or ferning) of saliva could be seen under a microscope during the periovulatory period. Increasing levels of estrogen and adrenocorticotropic hormone before ovulation stimulates the secretion of aldosterone, which regulates the electrolytes and fluid status in human body.^46^ Crystallization of NaCl produces the ferning appearance of saliva observed under a microscope.

A pocket microscope specifically designed for salivary ferning observation has been developed. A study conducted by Barbato et al. declared that salivary ferning correlated well to BBT and cervical mucus methods.^46^ It was later reported that salivary ferning can be detected in postmenopausal, pregnant, prepubertal women, and even in men. In women with regular menstruation, salivary ferning predicted ovulation with a sensitivity of only 53% correlated to ultrasonography and serum LH.^48^, ^49^ Guida et al. compared several methods to ultrasonography‐detected ovulation and found that positive salivary ferning test correlated to −1 to +1 days of actual ovulation in 42% of cases. However, a high percentage of uninterpretable patterns (58.7%) was reported.^41^ A recent study compared salivary ferning using a *Geratherm ovu control* microscope to a urinary LH test using *EXACTO* monitors and found a high correlation between these methods.^50^

To perform this test, women could use a small microscope with built‐in or removable slides. A drop of saliva may be applied to a slide, allowed to dry and inspected for ferning. The U.S. FDA announced that a positive test indicates that women may be near ovulation, but a negative test is unreliable for contraception.

Sex hormones were first measured in saliva by radioimmunoassay in 1978. Early publications focused on the correlation of salivary steroids to ovarian function,^19^, ^20^, ^51^, ^52^, ^53^, ^54^ but exact ovulation time was neither determined nor even estimated, partly because reliable methodology was not yet available. Based on the theory that increased circulating estrogens stimulate the breakdown of glycogen, Alagendran et al. reported that levels of sialic acid and glycosaminoglycans (CAG) increased during the ovulatory period.^55^ A change in urinary CAG level during the ovulatory phase was also demonstrated, as the ratio of urinary trypsin inhibitor/chondroitin sulphate peaked at menstrual cycle day 12.^56^

Recently, attention was given to examining salivary proteins for various diagnostic purposes. Protein concentration is at its highest during the ovulation phase. By single dimension SDS‐PAGE analysis, a 48 kDa protein was identified to exhibit predominantly during ovulatory phase.^57^ The author suggested that the protein level and the presence of 48 kDa band might be recognized as ovulation indicators. Further research is needed on this topic.

## 3 POC OVULATION DETECTION DEVICES

### 3.1 Urinary luteinizing hormone

**Figure 3:**
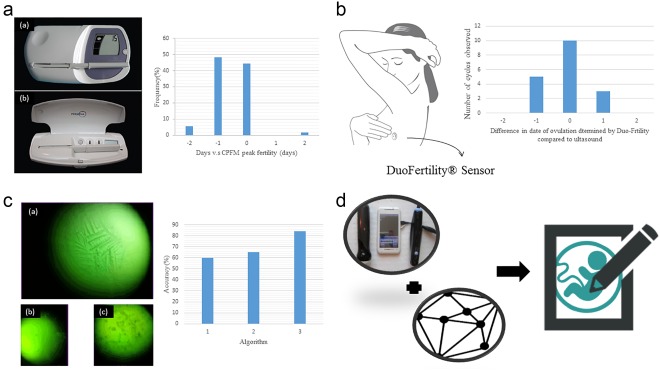
(a) The left graph is the photo of Clearblue Easy Fertility Monitor (a) and the Persona (b).^60^ The right bar chart is the Serum LH surge relative to CPFM peak fertility. The *X*‐axis −2 and 2 represents >1 day before or after CPFM peak fertility, respectively. Cycles with no CPFM peak fertility (*n* = 13), no serum LH surge day (*n* = 10), or neither (*n* = 1) are excluded from the table.^61^ (b) The left graph is the illustration of DuoFertility® Sensor worn by patients. The right bar chart is the correlation between ultrasound scans and DuoFertility® result.^62^ (c) The left graph is the result of ferning patterns by Knowhen Ovulation Monitoring System. (a) Fertile (b) preovulatory (c) postovulatory.^63^ The accuracy of three proposed algorithms. Algorithm 1 is binarizing + dark pixel density. Algorithm 2 is binarizing + dark pixel density + thinning. Algorithm 3 is binarizing + Hough transform + thinning + decision tree.^64^ (d) The illustration of possible combination of smartphone ultrasound device and imaging processing algorithms as an ovulation detection device^65^

Nowadays, many POC and over‐the‐counter ovulation detection devices are easily accessible for women planning conception or contraception. Most of these commercialized products determine ovulation by detecting urinary LH level. Several computerized fertility monitors have been used to detect urinary LH and estrone‐3‐glucoronide, which, if presented in urine, have also indicated ovulation.^58^ The manufacturer of the Clearblue easy fertility Monitor (*CEFM*) reported a significantly higher pregnancy rate during the first 2 cycles in women using its product compared to control groups (22.7% compared to 14.4%).^59^ (Figure 3a) Moreover, the same manufacturer developed an additional product, Clearblue DIGITAL Ovulation Test with Dual Hormone Indicator, which detects estrogen as well as LH. The manufacturer claims that this product can identify 4 or more fertile days with an accuracy rate of over 99%. Both monitors are digital immunoassays incorporating a disposable microspectrophotometer.^66^ Primarily used for contraceptive purposes, the *Persona* monitor, achieved a 94% correct‐use effectiveness.^67^, ^68^ (Figure 3a) The sensitivity of *CEFM* and *Persona* to accurately determine ovulation are 97% and 95.8%, respectively.^61^

### 3.2 Basal body temperature

In addition to urinary LH level detection, some commercialized POC devices adopt BBT monitoring methods to determine ovulation. OvuSense is a vaginal temperature sensor with a 99% claimed accuracy rate for ovulation date detection and an 89% accuracy rate for ovulation date prediction.^69^ In addition to measuring temperature intravaginally, some devices base their results on temperature measured elsewhere on the body. NaturalCycles, for example, which consists of thermometers and mobile application, is stated to misidentify only 0.05% of nonfertile days as fertile days.^70^ By measuring and recording temperature on a daily basis, the NaturalCycles application can detect ovulation and fertile days. Another product, DuoFertility® employs a small sensor worn under the arm that takes thousands of measurements throughout the day and night and reports temperature data to a server (Figure 3b). DuoFertility automatically recognizes ovulation through proprietary algorithms with 100% sensitivity.^62^

### 3.3 Salivary ferning and analysis

Some portable microscopes have been developed to detect ovulation via salivary ferning analysis. Among such devices, the manufacturers of the Knowhen Ovulation Monitoring System have shown a strong correlation between observed salivary ferning and ovulation among 22 patients (Log Odds ratio 7.64, *p* < .01, CI 4.26–11.02)^63^ (Figure 3c). Another device, Geratherm® ovu control, is reported to have a specificity of 78% and a sensitivity of 80%.^71^ Both of the abovementioned devices require patients to self‐evaluate salivary ferning. The agreement rate between saliva test carried out by patients and by laboratory staff is 89.4% with Geratherm® ovu control.^71^ To avoid potential self‐evaluation error WU, Hui‐Ching et al. introduced an automatic salivary ferning pattern recognition system for ovulation detection. They report an accuracy rate of 84% from 100 saliva samples.^64^

### 3.4 Smartphone‐based approaches

While promising, none of the above‐mentioned devices are robust. LH detection, detection, BBT monitoring, and salivary ferning analysis are easily influenced by other body conditions such as polycystic ovarian syndrome, abnormal increases in estrogen levels or fever.^72^ Some new technologies, including smartphone ultrasound apparatuses and novel software algorithms, may improve precision while keeping cost down. Considering the fact that transvaginal ultrasonography is the standard reference examination, smartphone ultrasound technology, which is now commercially available, may provide the most accurate at‐home ovulation detection results (Figure 3d). A fully developed imaging processing program may replace expertise in interpreting ultrasound scan results. Furthermore, imaging processing algorithms are available to improve the accuracy of salivary ferning pattern reading.^64^ Additionally, decision making algorithms can help detect ovulation by analyzing BBT patterns to increase the accuracy of ovulation detection by BBT analysis.^72^

### 3.5 Paper‐based approaches

Aside from smart‐phone based devices, paper‐based ovulation detection devices are full of potential. Paper has been successfully used for biological assays, such as ELISA^73^ and cell assays.^74^ Paper is economical, prevalent, disposable, and suitable for large‐scale manufacture.^75^ Paper‐based ovulation detection devices based on LH detection, such as One Step® Ovulation & Pregnancy Test Kit Strips, are currently available.

### 3.6 Cervicovaginal fluid

Besides, a new developed cotton‐based cervicovaginal fluid collecting device may offer another solution. Cheng et al. proposed a readily applicable cervicovaginal fluid collection device, which saves cervicovaginal fluid for later diagnosis. It is proven to successfully determine pathogen infection and to diagnose the presence of female genital cancer.^60^ This device may collect cervicovaginal fluid for determining ovulation by cerival mucus detection, which is the least expansive but effective method. Furthermore, coupling cervicovaginal fluid collecting device with thermometers makes a simple point‐of‐care ovulation detection applying symptothermal method.

## 4 SUMMARY AND PERSPECTIVES

Most of the currently available methods to detect ovulation were developed decades ago (Table 1). Research tests for the methods described here were limited to small sample sizes and subjects of limited cultural background (most studies were performed in European countries in less than 100 women with less than 1,000 cycles). Even fewer studies examined or included women having irregular menstruation, which is characterized by less predictable ovulation timing. Such women would particularly benefit from improved ovulation predictors. While differences in environmental hormones and dietary habits may considerably influence hormone status in women, re‐examination by large database study may change our view regarding these available methods.

**Table 1:** Features of currently available methods to detect ovulation

	Cost	Accuracy	Accessibility	Invasion	Detect before ovulation	Features/disadvantage
POC methods available						
Urinary LH	Low cost of kits	High (97%)	High (OTC)	No	Yes	Repeated purchases of kits
Computerized monitor (urinary LH + E1‐3‐G)	Moderate cost of device	High (95.8–97%)	High (OTC)	No	Yes	Evidence to improve pregnancy rate Repeated purchases of sticks
Basal body temperature	Low cost of thermometer	Low (22.1%)	High	No	No	Not easily interpreted Affected by environmental factors
Cervical mucus	No cost	Moderate (48–76%)	High	No	Yes	Unable to perform while vaginal infection
Salivary ferning	Low cost of kits	Moderate (42–53%)	High (OTC)	No	Yes	High percentage of unpredictable result
POC methods unavailable						
Transvaginal ultrasound	High	High (standard reference examination)	Low (performed by physician)	Yes (introduce vaginal probe)	Yes	May be uncomfortable during exam
Serum progesterone	N/A^a^	High (89.6%)	Low (need laboratory)	Yes (venipuncture)	No	Confirms ovulation
Urinary PDG	N/A^a^	High (92.2%)	Low (need laboratory)	No	No	Confirms ovulation

a These two exams are not commonly performed. The cost may vary in different country.

E1‐3‐G = estrone‐3‐glucoronide; LH = leutinizing hormone; N/A = not applicable; OTC = over‐the‐counter; PDG = pregnanediol 3‐glucuronide.

An ideal method to detect ovulation should be (a) noninvasive, (b) inexpensive, (c) easily available and easy to use (as a POC method), (d) precise in determining ovulation, and (e) precise in determining the fertility window. None of the aforementioned methods fits all of these features. However, with modern technology, a combination of different methods may be incorporated into one small pocket machine for computerized analysis. A better understanding of physical and hormonal changes during ovulation and improvements in biotechnology may help develop additionally useful and accurate methods to detect ovulation.

## CONFLICT OF INTERESTS

None of the authors have any conflict of interest to declare regarding the manuscript.

## AUTHOR CONTRIBUTIONS

Design and conduct of the study: T.‐C.C. and C.‐M.C. Writing of the article: H.‐W. S., Y.‐C. Y., T.‐Y. W., T.‐C.C., and C.‐M.C. Final approval of the article: H.‐W. S., Y.‐C. Y., T.‐Y. W., T.‐C.C., and C.‐M.C. Literature search: H.‐W. S., Y.‐C. Y., and T.‐Y. W. Preparation of figures: H.‐W. S., Y.‐C. Y., and T.‐Y. W. Administrative, technical and logistical support: H.‐W. S., Y.‐C. Y., T.‐Y. W., T.‐C.C., and C.‐M.C.
